# Potential use of sodium glucose co-transporter 2 inhibitors during acute illness: a systematic review based on COVID-19

**DOI:** 10.1007/s12020-024-03758-8

**Published:** 2024-03-06

**Authors:** Carmen Tisch, Eleni Xourgia, Aristomenis Exadaktylos, Mairi Ziaka

**Affiliations:** 1 Department of Internal Medicine, Thun General Hospital, Thun, Switzerland; 2 Department of Cardiology, Inselspital, University Hospital, University of Bern, 3008 Bern, Switzerland; 3 Department of Emergency Medicine, Inselspital, University Hospital, University of Bern, Bern, Switzerland

**Keywords:** Acute Illness, COVID-19, Diabetic Ketoacidosis, Intensive Care Unit, Mechanical Ventilation, SGLT-2 Inhibitors

## Abstract

**Objective:**

SGLT-2i are increasingly recognized for their benefits in patients with cardiometabolic risk factors. Additionally, emerging evidence suggests potential applications in acute illnesses, including COVID-19. This systematic review aims to evaluate the effects of SGLT-2i in patients facing acute illness, particularly focusing on SARS-CoV-2 infection.

**Methods:**

Following PRISMA guidelines, a systematic search of PubMed, Scopus, medRxiv, Research Square, and Google Scholar identified 22 studies meeting inclusion criteria, including randomized controlled trials and observational studies. Data extraction and quality assessment were conducted independently.

**Results:**

Out of the 22 studies included in the review, six reported reduced mortality in DM-2 patients taking SGLT-2i, while two found a decreased risk of hospitalization. Moreover, one study demonstrated a lower in-hospital mortality rate in DM-2 patients under combined therapy of metformin plus SGLT-2i. However, three studies showed a neutral effect on the risk of hospitalization. No increased risk of developing COVID-19 was associated with SGLT-2i use in DM-2 patients. Prior use of SGLT-2i was not associated with ICU admission and need for MV. The risk of acute kidney injury showed variability, with inconsistent evidence regarding diabetic ketoacidosis.

**Conclusion:**

Our systematic review reveals mixed findings on the efficacy of SGLT-2i use in COVID-19 patients with cardiometabolic risk factors. While some studies suggest potential benefits in reducing mortality and hospitalizations, others report inconclusive results. Further research is needed to clarify optimal usage and mitigate associated risks, emphasizing caution in clinical interpretation.

## Introduction

SGLT2i represents a novel category of oral agents designed to lower glucose levels in individuals with DM-2. Additionally, they serve as adjunct therapy for individuals with DM-1 who are overweight and not adequately responsive to insulin. Gliflozins operate by inhibiting SGLT-2, a transporter expressed in the early segment of the proximal renal tube, thereby diminishing renal glucose reabsorption. The introduction of this drug class has transformed the landscape of diabetes management. This transformation is not only attributed to their efficacy in reducing blood glucose levels with minimal hypoglycemic risk but is primarily underscored by their noteworthy cardiorenal protective properties. Compelling evidence from recent comprehensive cardiovascular outcome trials has firmly linked SGLT2i treatment to substantial decreases in the risk of major cardiovascular (CV) events, hospitalization due to heart failure (HF), CV mortality, and renal complications [1–3]. Indeed, in 2019, the DAPA-HF trial elucidated a significant clinical advantage associated with dapagliflozin in individuals with heart failure and reduced ejection fraction, irrespective of the concomitant presence or absence of diabetes resulting to a pivotal juncture for many, culminating years of conjecture [4, 5].

The first documented infections with the novel SARS-CoV-2 occurred in December 2019 [6–8], approximately six months following the publication of the DAPA-HF study. Since the beginning of the pandemic, intensive research has been conducted, on the one hand on risk factors for a severe course, and on the other hand on therapy during Coronavirus Disease 2019 (COVID-19). The clinical spectrum of the disease ranges from asymptomatic to severe courses with pneumonia, respiratory failure to acute respiratory distress syndrome (ARDS), multiple organ failure (MODS), and fulminant myocarditis [6–8]. Risk factors of the disease include older age and co-existed comorbidities such as cardiovascular diseases and hypertension, diabetes mellitus (DM) type 1 and DM-2, obesity, and chronic kidney disease (CKD) with these conditions being independently associated with increased in-hospital mortality [9–12]. Multiple pathways have been postulated to be involved in severe COVID-19 including systemic inflammation and dysregulation of pro-inflammatory cytokines (e.g., interleukin (IL)-1, IL-2, IL-6), which can culminate in a cytokine storm [6], increased production of free radicals, and enhanced oxidative stress [13].

In addition to the primary injury, the pathophysiology of severe COVID-19 involves systemic inflammation-associated secondary events such as hypotension, left ventricular dysfunction, arrhythmia, thromboembolic complications, and destabilization of vascular plaques [6, 14]. Hence, given that inflammation constitutes a pathophysiologic mechanism amenable to modification, a fundamental objective in the management of COVID-19 is to minimize or inhibit systemic inflammation and the release of pro-inflammatory cytokines.

DKA is an infrequent yet severe complication of type 2 diabetes, characterized by a notably high case fatality rate [15]. The absolute risk of DKA in extensive, prospective randomized clinical trials involving individuals with type 2 diabetes using SGLT-2i has been demonstrated as minimal. However, the relative risk is elevated in those assigned to SGLT-2i compared to those receiving a placebo. In individuals without diabetes but prescribed SGLT-2i for conditions such as heart failure or CKD, the risk of DKA mirrors that of the placebo group [16]. Consensus guidelines, however, advise against the use of SGLT-2i in cases of serious illness and do not recommend their routine in-hospital administration [17]. Throughout the COVID-19 pandemic, instances of DKA have been documented in individuals hospitalized with COVID-19 [18]. This strategy was based on the risk of DKA in critically ill patients [17, 19] and the findings that COVID-19 is associated with hyperglycemic emergencies, including DKA and long-lasting ketosis, in people with DM-2 and COVID-19 [18]. Moreover, the increased expression of angiotensin-converting enzyme 2 (ACE-2) in lungs, brain, heart, kidney, and veins in patients taking SGLT-2i [20, 21], which, as a receptor for SARS-CoV-2, could, in theory, lead to higher risk of infection [22], represent further counterarguments. Nevertheless, recent data propose potential favorable effects of SGLT-2i in the context of acute illness with COVID-19, indicating no increase in adverse events and low rates of non-severe DKA [23, 24].

Indeed, several meta-analyses have demonstrated a potential benefit regarding morbidity and mortality in patients suffering from COVID-19 in SGLT-2i users with DM-2 [25–29]. Moreover, as indicated by experimental models, treatment with SGLT-2i offers a beneficial approach in order to blunt systemic inflammation and immune responses, reduce oxidative stress and obesity-associated inflammation, and modulate renin-angiotensin system activity [30–33]. Therefore, based on the encouraging study results and given the beneficial effects of SGLT-2i, discontinuation of SGLT-2i during acute illness should be further evaluated [34, 35].

The existing meta-analyses have primarily focused in patients with DM-2. However, the indications for SGLT-2i are becoming broader due to the encouraging study situation with more and more patients being treated with this class of drugs. In addition to the blood glucose-lowering effect, the nephroprotective [1], cardioprotective [4, 36], and antiinflammatory effects [37, 38] seem also essential. Given the low incidence of DKA in cardiovascular outcome trials and in hospitalized patients with DM-2 [16], coupled with the abundance of pertinent, recently published studies and existing uncertainties surrounding the administration of SGLT-2 inhibitors during acute illness, this systematic review seeks to furnish evidence on the utilization of SGLT-2i in patients experiencing acute illness, with a particular focus on SARS-CoV-2 infection.

## Methodology

### Protocol and registration

The present systematic review adheres to the Preferred Reporting Items for Systematic Reviews and Meta-Analyses statement [39]. Our protocol was pre-registered with PROSPERO (CRD42023457993) and is accessible online.

### Inclusion and exclusion criteria

We included randomized controlled trials and observational cohort studies in our analysis, encompassing data related to the use of SGLT-2i in patients with COVID-19 and providing information on all-cause mortality and/or morbidity outcomes. Both peer-reviewed papers and preprints were deemed eligible, while case reports and case series involving fewer than five patients were excluded.

### Outcomes of interest

The primary outcome was to investigate the mortality of patients with COVID-19 while receiving SGLT-2i. Secondary outcomes included the effect of SGLT-2i use in patients with SARS-CoV-2 on systemic inflammation, hospitalization, ICU admission, need for mechanical ventilation, effect on renal function, and prevalence of adverse events, particularly AKI and DKA.

### Search strategy

Two authors (CT and MZ) independently conducted the literature search. We systematically searched PubMed and Scopus in order to explore all available clinical studies on the topic with the search phrase: (“SARS-CoV-2” OR “COVID-19” OR “Corona Virus”) AND (“SGLT-2” OR “Sodium-glucose transporter-2” OR “antidiabetic*” OR “anti‐diabetic*” OR “medication*” OR “treatment” OR “drug*”) AND (“diabetes” OR “T2DM”). We also conducted a search in the gray literature (i.e., preprint serversmedRxiv and Research Square and Google Scholar) by using the same search phrase. Another search was conducted in the reference lists of all identified reports and articles for additional studies We retrieved all relevant articles on adult human subjects up to October 30th, 2023, with no language restrictions.

### Data extraction

The titles and abstracts of studies acquired through the search strategy and additional sources underwent independent screening by three authors (CT, EX, and MZ) to identify those potentially aligning with the aforementioned inclusion criteria. Data extraction from each study was independently conducted by two authors (CT and MZ) using a customized format. Any disagreements regarding the eligibility of studies were resolved through discussions among the authors.

A standardized form was employed for the systematic extraction of data from the encompassed studies, facilitating the evaluation of study quality and the synthesis of evidence. The extracted information encompassed publication particulars (authors, year), geographical location, study type, clinical attributes (comorbidities such as DM, hypertension, ischemic cardiac disease, heart failure, kidney injury, DKA), and outcomes. Discrepancies were addressed through dialog or, when required, through consultation with other authors. Correspondence with the original study authors was initiated to seek clarifications or obtain supplementary information.

### Assessment of methodological quality

The articles selected for retrieval underwent evaluation by two independent authors (CT, EX) for methodological quality using the Newcastle-Ottawa Quality Assessment Scale for Cohort Studies [40] before inclusion in the review. Any discrepancies between the authors during the appraisal process were resolved through discussion involving all the authors. Further details are available in the Supplementary Material.

## Results

**Fig. 1:**
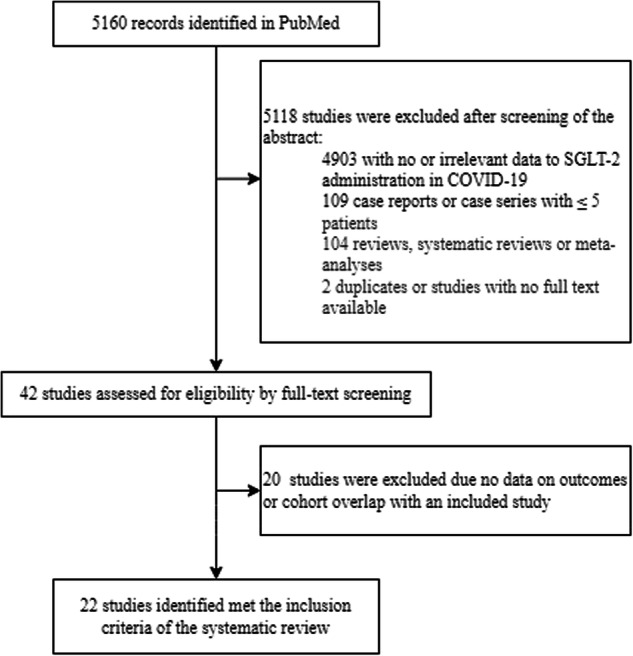
The PRISMA flow diagram for the study selection

Out of the 5160 pertinent citations identified and screened, 22 studies were chosen for a comprehensive review following an assessment of their abstracts. These 22 studies were included in the final evaluation for potential data extraction (Fig. 1). The baseline characteristics of the studies investigating the use of SGLT-2i in patients with COVID-19 incorporated into the systematic review are detailed in Table 1. A summary of the results from the risk of bias assessment for the included studies can be found in the supplementary appendix (Table S1).

**Table 1:** Studies of SGLT-2i use in patients with COVID-19

Author	Country	Design	Study’s population	Main finding
Kosiborod et al. [23]	Multiple countries	Randomized controlled trial	Patients with cardiometabolic risk factors	Dapagliflozin intake during COVID-19 in patients with cardiometabolic risk factors has no significant effect on organ dysfunction or death, nor does it improve recovery. AKI occurred in 3.4% of the study population and DKA in 0.3%.
Bhatt et al. [24]	Multiple countries	Prospective, Double-Blind, Event-Driven Clinical Study	HFmrEF/HFpEF	Dapagliflozin reduced cardiovascular death/worsening HF events in patients with chronic heart failure with HFmrEF/HFpEF, when censoring participants at COVID-19 diagnosis and pandemic onset No DKA or major hypoglycaemic events within 30 days of COVID-19.
Li et al. [33]	China	Experimental Study	Experimental SARS-CoV-2-mediated cardiorenal injury	Downregulation of apelin and ACE2 and upregulation of SGLT2, endothelin-1 and pro-inflammatory cytokines in post-MI HF rats, AKI and diabetic mice contribute to SARS-CoV-2-mediated cardiorenal injury
Khunti et al. [34]	England	Observational Cohort Study	DM-2	DM-2 patients taking SGLT-2i have a lower mortality risk than those not prescribed.
Khunti et al. [35]	England	Retrospective Cohort Study	DM-2	No increased risk of DKA or in-hospital mortality associated with prescription of SGLT-2i in patients with DM-2.
Sainsbury et al. [53]	England	Retrospective Cohort Study	DM-2	No elevated risk of developing COVID-19 in patients with DM-2 while SGLT-2i intake compared to DPP-4 inhibitors.
Kahkoska et al. [41]	USA	Observational Cohort Study	Diabetic patients, no further specified	Diabetic patients with COVID-19, using SLGT-2i, had a lower rate of adverse outcomes and lower mortality compared to DPP4i-user.
Shestakova et al. [42]	Russia	Observational Cohort Study	DM-2	Patients with DM-2 taking SGLT-2i have a lower risk of death when having COVID-19.
Mroueh et al. [83]	Multiple countries	Prospective in vitro Study	Patients with acute, subacute, and long COVID-19	Empagliflozin improved the deleterious impact of COVID-19 on endothelial cells.
Ramos-Rincón et al. [46]	Spain	Observational Cohort Study	DM-2	No effect of SGLT-2i use before admission between non-survivor and survivor geriatric patients with DM-2.
Pérez-Belmonte et al. [45]	Spain	Observational Cohort Study	DM-2	A lower in-hospital death rate was highlighted in DM-2 patients under combined therapy of metformin plus SGLT-2i. Combined therapy showed no significant association with mortality, need of intensive care unit admission, mechanical ventilation, in-hospital complications, or long-time hospital stays.
Min et al. [47]	USA	Retrospective Cohort Study	DM-2	No elevated mortality and risk of hospitalization in COVID-19 patients with DM-2 while SGLT-2i intake compared to other antidiabetic substances. AKI occurred in 43.4% of the patients and DKA in 9.4%.
Israelsen et al. [51]	Denmark	Retrospective Cohort Study	Diabetes, not further specified	The use of GLP‐1 RAs or DPP‐4i compared with SGLT‐2i in patients with diabetes was not associated with decreased risk of hospital admission.
Kim et al. [48]	South Korea	Retrospective Observational Cohort Study	Diabetes, not further specified	Prior use of SGLT-2i did not impair disease severity and mortality in diabetic patients with COVID-19
Sourij et al. [49]	Austria	Prospective and Retrospective Cohort Study	Diabetes and prediabetes, not further specified	Preadmission use of SGLT-2i did not affect in-hospital mortality in patients with diabetes and prediabetes.
Wander et al. [43]	USA	Retrospective Cohort Study	Diabetes, not further specified	SGLT-2i use in diabetic patients was associated with lower odds of hospitalization and risk of death.
Yeh et al. [50]	USA	Retrospective Cohort Study	DM-2	In patients with DM-2 diabetes prior use of SGLT-2i was not associated with hospitalization, ICU admission, intubation or death.
Sandhu et al. [52]	USA	Retrospective Cohort Study	Mixed population incl. patients with cardiometabolic risk factors	The combination metformin empagliflozin showed protective effects against COVID-19 hospitalization. Empagliflozin alone was not associated with a lower risk of hospitalization.
RECOVERY Collaborative Group [57]	Multiple countries	Randomized Controlled, Open-Label Study	Hospitalized patients with COVID-19	In adults hospitalized with COVID-19, empagliflozin was not associated with reductions in 28-day mortality, duration of hospital-stay, or risk of progressing to invasive MV or death. DKA was observed in 0.2% of the patients.
Foresta et al. [44]	Italy	Retrospective Cohort Study	DM, not further specified	Trend (not statistically significant) in risk reduction for total mortality and hospitalization for DM patients with COVID-19 undes SGLT-2i compared with nonusers. Statistical significance for in-hospital mortality in users of SGLT-2i compared with nonusers
Salgado-Barreira et al. [55]	Spain	Population-based Case-control Study	Mixed population incl. patients with cardiometabolic risk factors	Use of dapagliflozin prior to SARS-CoV-2 infection was not associated with an increased risk of hospitalization, ICU-admission, mortality or progression to severe COVID-19. However, it was associated with an increased risk of susceptibility to COVID-19. Dapagliflozin reduced the risk of progression to severe COVID-19 by 35% (not statistically significant).
Dalan et al. [54]	Singapore	Retrospective Observational Cohort Study	DM, not further specified	SGLT-2i use was associated with a marginally lower risk of MV in patients with DM.

*ACE2* Angiotensin converting enzyme 2, *AKI* Acute kidney injury, *COVID-19* Corona virus disease 2019, *DKA* Diabetic ketoascidosis, *DM* Diabetes mellitus (Type II), *DPP-4* Dipeptidylpeptidase 4, *GLP‐1* RA Glucagon-like peptide 1 receptor agonist, *HF* Heart failure, *HFmrEF* Heart failure with mildly reduced ejection fraction, *HFpEF* Heart failure with preserved ejection fraction, *ICU* Intensive care unit, *MV* mechanical ventilation, *post-MI HF* Post myocardial-injury heart failure, *SARS-CoV-2* Severe acute respiratory syndrome coronavirus type 2

### Diabetes mellitus patients

#### Mortality

Four studies [34, 41–43] reported that DM-2 patients taking SGLT-2i had a lower total mortality risk than those not prescribed. One study [44] could demonstrate a trend in risk reduction for total mortality and hospitalization for DM patients with COVID-19 under SGLT-2i compared with nonusers without however reaching statistical significance. One study [44] highlights that DM infected with COVID-19 patients using SGLT-2i have a significantly lower in-hospital mortality compared to nonusers. Moreover, one study [45] showed a lower in-hospital death rate in DM-2 patients under combined therapy of metformin plus SGLT-2i. Furthermore, six studies found no elevated mortality in COVID-19 DM patients while taking SGLT-2i [16, 46–50].

#### Hospitalization

Two studies [43, 44] found a reduced risk of hospitalization in COVID-19 patients with DM-2 while taking SGLT-2i. However, the study of Foresta et al. [44] does not reach statistical significance regarding risk of hospitalization [44]. Three studies [47, 51, 52] demonstrated a neutral effect on risk of hospitalization.

#### Susceptibility to COVID-19 infection

In a retrospective cohort study, Sainsbury et al. (2021) found that the use of SGLT-2i in DM-2 patients did not elevate the risk of developing COVID-19 [53].

#### Intensive care unit admission and mechanical ventilation

In patients with DM-2, prior use of SGLT-2i was not associated with intensive care unit (ICU) admission and need for mechanical ventilation (MV) [50]. Similarly, combined therapy with metformin and empagliflozin showed no significant association to ICU admission and MV [45]. In contrast, the study of Dalan et al. [54] highlighted that SGLT-2i use was associated with a marginally lower risk MV in patients with DM [54].

### Use of SGLT-2i in patients with cardiometabolic risk factors and/or heart failure

One multicenter, prospective, double-blind, event-driven clinical study found that dapagliflozin reduced cardiovascular death/worsening heart failure events in patients with chronic heart failure with HFmrEF/HFpEF when censoring participants at COVID-19 diagnosis [24]. Moreover, the combination of metformin and empagliflozin exhibited protective effects against COVID-19 hospitalization in patients with cardiometabolic risk factors. However, empagliflozin alone was not associated with a lower risk of hospitalization [52]. Likewise, the use of dapagliflozin before contracting SARS-CoV-2 did not demonstrate an elevated risk of severe outcomes. While an increased susceptibility to COVID-19 was noted, dapagliflozin showed a trend in reducing the risk of progressing to severe COVID-19, although this trend did not reach statistical significance [55]. These findings are further supported by experimental evidence showing that downregulation of apelin and ACE2 and upregulation of SGLT-2, endothelin-1, and pro-inflammatory cytokines contribute to SARS-CoV-2-mediated cardiorenal injury in post-myocardial infarction heart failure rats, acute kidney injury (AKI), and diabetic mice [56]. In contrast, the DARE-19 study failed to demonstrate a benefit in patients with cardiometabolic risk factors. Specifically, dapagliflozin intake during COVID-19 in patients with cardiometabolic risk factors showed no significant effect on organ dysfunction or death [23]. Similarly, in a multicountry randomized controlled study, there was no association found between empagliflozin and reductions in 28-day mortality, duration of hospital stay, or the risk of advancing to invasive MV or death in adults hospitalized with COVID-19 [57].

### SGLT-2i and acute kidney injury in patients with COVID-19

Only a few studies reporting the effects of SGLT-2i on renal function in patients with COVID-19 could be identified. The DARE-19 study demonstrated that dapagliflozin intake during COVID-19 in patients with cardiometabolic risk factors was associated with the development AKI in 3.4% of the study population [23]. Moreover, the RECOVERY trial did not identify any discernible difference in the occurrence of acute kidney injury, defined as an increase in the pre-randomization creatinine concentration of ≥50%, or the requirement for renal dialysis or haemofiltration between the treatment groups (empagliflozin vs. standard care, 6.3 vs. 6.1%, respectively) [57].

However, a significantly higher AKI occurrence is reported by Min and co-workers [47] in a retrospective cohort study on patients with DM-2 (43.4%) [47]. Nevertheless, experimental findings indicate that the downregulation of apelin and ACE2, coupled with the upregulation of SGLT-2, endothelin-1, and pro-inflammatory cytokines, play a contributory role in the cardiorenal injury mediated by SARS-CoV-2 in rats with post-myocardial infarction heart failure, as well as in mice with AKI and diabetes [56].

### SGLT-2i and diabetic ketoacidosis in patients with COVID-19

Based on the provided studies, the association between the use of SGLT-2i and the risk of DKA in patients with DM-2 and in patients with cardiometabolic risk factors and/or heart failure during COVID-19 was assessed. Three studies collectively indicate a consistent finding that the prescription of SGLT-2i, such as dapagliflozin and empagliflozin, is not linked to an elevated risk of DKA in individuals with DM-2 and in patients with cardiometabolic risk factors and/or heart failure as well [23, 24, 35, 57]. Furthermore, the studies suggest that SGLT-2i do not contribute to a higher likelihood of in-hospital mortality in individuals with DM-2 during COVID-19, as evidenced by the findings of Khunti et al. [35]. In the RECOVERY trial, the occurrence of metabolic complications was comparable between the empagliflozin and standard care groups, with reported cases of DKA in five (0.2%) versus two (0.1%) patients, respectively [57]. However, the study of Min et al. [47] demonstrates an incidence of DKA associated with the use of SGLT-2i in 9.4% of DM-2 patients with COVID-19 [47].

## Discussion

Observations derived from good structured randomized controlled trials indicate that SGLT-2i inhibitors exhibit a mitigating effect on the progression of cardiovascular and kidney diseases, irrespective of the presence or absence of diabetes [58]. Furthermore, in individuals with CKD and notable albuminuria, the administration of the SGLT-2i dapagliflozin has demonstrated a reduction in the risk of precipitous declines in kidney function, characterized by a twofold increase in serum creatinine levels between two consecutive study visits with a median time-interval of 100 days [59]. However, typically, the control of elevated blood glucose levels in hospitalized patients relies on insulin therapy. The abstention from oral agents during hospitalization is grounded in their diminished hypoglycemic efficacy relative to insulin, potential interactions with concurrently prescribed medications, and various safety concerns associated with altered pharmacokinetics, particularly in instances of renal or hepatic dysfunction [60]. Issues of concern encompass identified correlations between SGLT-2i and an elevated risk of DKA, hypovolaemia [60], disruptions in electrolyte and acid–base balance [61], urinary tract infections [62], as well as an early decrease in glomerular filtration rate induced by tubuloglomerular activation and reduced intraglomerular pressure [63]. Indeed, observational data from real-world settings indicate that the occurrence of DKA continues to be a point of concern in the context of using SGLT-2i [64] especially if specific medical conditions as for example acute infectious illness, urgent surgical procedures, or extended periods of fasting, co-exist [3].

Cytokine storm observed in patients with SARS-CoV-2 denotes an aberrant hyperactivation of the immune system characterized by dysregulated proinflammatory cytokine production. This dysregulation precipitates an excessive infiltration of immune cells into pulmonary tissues, inducing consequential tissue damage. Furthermore, this immune cell infiltration may extend to diverse tissues and organs, thereby engendering dysfunction across multiple organ systems. Key cytokines implicated in disease severity encompass tumor necrosis factor α (TNF-α), interferon γ (IFN-γ), IL-6, IL-1β, granulocyte-macrophage colony-stimulating factor, and granulocyte colony-stimulating factor indicating that the judicious management of the hyperinflammation assumes paramount importance in the therapeutic intervention for COVID-19 disease [65]. Demonstrating efficacy in patients with severe COVID-19, corticosteroids, IL-6 inhibitors, and Janus kinase inhibitors [66] have been established as agents capable of reducing mortality [57] underscoring the modifiable nature of inflammation and the potential for improvement in clinical outcomes through anti-inflammatory therapeutic interventions.

In addition to their glucose-lowering properties, SGLT-2i have been observed to contribute to the establishment of an anti-inflammatory environment through various physiological mechanisms as for example the attenuation of the activation of nucleotide-binding domain-like receptor protein 3 (NLRP3) inflammasome leading to elimination of the production and expression of proinflammatory cytokines, including IL-1β [38], IL-1, IL-6, and TNF-α [67]. Additionally, they hinder glycolysis, a pathway utilized by respiratory pathogens, and promote lipolysis, diminish oxidative stress, alongside enhancing endothelial function and oxygen-carrying capacity and tissue hypoxia through elevation of the erythropoietin concentrations [3]. The beneficial effects of SGLT-2i on inflammatory cascades may be of significant importance in patients underwent MV, which is also associated with the development of severe inflammatory reactions [68–70]. Furthermore, SGLT2i appears to enhance the expression of ACE2 receptors, consequently elevating Angiotensin 1–7 levels, known for their protective effects against COVID-19-related ARDS. Moreover, these inhibitors may potentially mitigate myocarditis, reduce the risk of arrhythmias, impede heart failure progression, and attenuate kidney injury in affected individuals [71]. Nevertheless, considering the potential adverse events such as DKA, hypotension and hypovolaemia, along with genital mycotic infections that may be exacerbate during acute illness [72], and given the wealth of pertinent recently published studies and the prevailing uncertainties regarding the use of SGLT-2i during acute illness, this systematic review aims to provide evidence regarding the application of SGLT-2i in patients undergoing acute illness, using as model patients with SARS-CoV-2 infection.

### COVID-19-induced cytokine storm and SGLT-2 Inhibitors

Especially patients with DM-2, but also those with CKD as well as cardiovascular disease, experience an increase in IL-6 levels due to low-grade inflammation [73]. Low-grade systemic inflammation is associated with elevated uric acid and therefore linked with elevation of IL-6, C-reactive protein and TNF-α in the bloodstream [74]. All three underlying diseases are associated with an increased risk of severe COVID-19 progression [75]. Indeed, Chia et al [76] demonstrated that individuals from the general population with elevated IL-6 levels were significantly more likely to develop HFpEF than those with normal levels [76]. However, as is well known, all are also indications for SGLT-2i use. SGLT-2i are thought to decrease lactate accumulation by reversing the acid-base cytokine balance and preventing decrease of cytosolic pH. This results in inhibition of inflammatory pathways and subsequent cytokine storm, which in turn could damage the cells [77].

It has already been scientifically shown that SGLT-2i-induced lowering of the low-grade inflammation leads to a subsequent lowering of IL-6 and TNF-α levels in the blood [78–81]. Thus, the guideline-appropriate use of SGLT-2i in patients also appears reasonable in the acute phase of Sars-Cov-2 infection in the synopsis of the available studies. After searching large databases, no studies could be found that examined cytokine-levels when SGLT-2i were taken in critically ill patients. Given the available data, lower levels and thus a lowered inflammatory response would be conceivable. However, this effect has not been clinically demonstrated at this time.

A single prospective in vitro study was identified, focusing on the potential anti-inflammatory and protective properties of empagliflozin in the context of patients suffering from COVID-19. The central hypothesis of this study postulated that the inflammatory response observed in COVID-19 patients may contribute to endothelial dysfunction by means of upregulated, redox-sensitive SGLT-2 expression. Consequently, the investigation aimed to scrutinize the protective effects of inhibiting SGLT-2 with empagliflozin. This research entailed the collection of human plasma samples from three distinct cohorts, namely, patients with acute, subacute, and chronic COVID-19 conditions (*n* = 100), individuals without COVID-19 but exhibiting cardiovascular risk factors (*n* = 50), and a control group comprising healthy volunteers (*n* = 25). Porcine coronary artery endothelial cells were subjected to plasma exposure at a concentration of 10%. To evaluate the study’s outcomes, a suite of analyses was conducted, including Western blot assessments and immunofluorescence staining to gauge protein expression levels, quantitative reverse transcription-polymerase chain reaction to measure mRNA expression, and dihydroethidium staining to quantify oxidative stress. The research also encompassed the assessment of platelet adhesion, aggregation, and thrombin generation. The findings from this investigation unveiled heightened plasma levels of key proinflammatory cytokines, including interleukin IL-1β, IL-6, TNF-α, monocyte chemoattractant protein-1, and soluble intercellular adhesion molecule-1 in COVID-19 patients. Notably, exposure of ECs to COVID-19 plasma characterized by elevated proinflammatory cytokine levels (specifically, IL-1β, IL-6, and TNF-α) precipitated a redox-sensitive upregulation of SGLT-2 expression. This molecular response, in turn, fostered endothelial dysfunction, senescence, NF-κB activation, inflammation, platelet adhesion and aggregation, von Willebrand factor secretion, and thrombin generation. Crucially, the study demonstrated that the stimulatory effect of COVID-19 plasma could be attenuated through the use of neutralizing antibodies against proinflammatory cytokines and empagliflozin. In conclusion, the authors deduced that, in patients afflicted with COVID-19, proinflammatory cytokines induced the redox-sensitive upregulation of SGLT-2 expression in endothelial cells [82], which subsequently led to endothelial injury, senescence, platelet adhesion, aggregation, and thrombin generation. Consequently, the inhibition of SGLT-2 via empagliflozin presents itself as a promising strategy for restoring vascular homeostasis in the context of COVID-19 [83].

### SGLT-2 inhibitor in patients with diabetes mellitus and COVID-19

Individuals with DM-2 exhibit an elevated likelihood of hospitalization and face an augmented risk of severe outcomes and mortality [82] associated with COVID-19, as outlined by Zhang and co-workers [84]. Specifically, the risk for severe pneumonia is amplified by 2.3-fold, with a corresponding 2.5-fold increase in mortality [85]. This heightened susceptibility can be primarily attributed to two interconnected mechanisms: Firstly, a substantial proportion of patients with DM-2 are characterized by overweight. This adiposity contributes to an increased prevalence of cytokines, released by resident macrophages, adipocytes, and endothelial cells [86]. Consequently, the preexisting state of chronic inflammation is exacerbated during SARS-CoV-2 infection, culminating in a cytokine storm [87]. Importantly, the administration of SGLT-2i has been associated with a reduction in body weight [88], which, in turn, contributes to the amelioration of chronic inflammation in obese patients [89]. Conversely, heightened blood glucose concentrations prompt heightened expression of ACE2 in the pulmonary system [87] facilitating the internalization of the virus into the body [90]. To mitigate the potential severity of a COVID-19 infection, precise control of blood glucose levels is strongly recommended. On the other hand, elevated blood glucose levels stimulate increased expression of ACE-2 in the lung [87], which mediates the internalization of the virus to the body [90]. To avoid severe COVID-19 courses as far as possible, a tight adjustment of blood glucose levels is strongly recommended. Notably, the association between elevated HbA1c levels and increased mortality during COVID-19 has been established [91]. The higher the HbA1c-levels, the higher the mortality during COVID-19 with the levels of HbA1c presenting a linear correlation with mortality [91].

Existing evidence indicates that individuals with DM-2 undergoing treatment with SGLT-2i while suffering from COVID-19 manifest reduced mortality rates [28, 34, 41, 42]. According to Shestakova’s et al. [42], the favorable impact of SGLT-2i on the course of COVID-19 is ascribed to their capacity for diminishing oxidative stress, imparting an antioxidant effect, and modulating endothelial function [42]. Khunti et al. [34] observe a more pronounced beneficial effect in patients with diabetes mellitus and elevated body mass index or a history of cardiovascular disease during COVID-19, attributing this effect to the heightened cardio- and renoprotective properties intrinsic to this drug class [34].

We found that individuals with DM-2 taking SGLT-2i exhibit a lower risk of mortality, as reported by multiple studies [34, 41–43]. Foresta et al. (2023) suggests a trend towards reduced mortality and hospitalization for DM patients with COVID-19 under SGLT-2i, with significant in-hospital mortality reduction, particularly when combined with metformin [44]. Hospitalization risk reduction in DM-2 patients with COVID-19 under SGLT-2i was noted in studies by Wander et al. [43] and Foresta et al. [44] (not statistically significant) [43, 44]. However, three studies [51, 52] showed a neutral effect on hospitalization risk. The susceptibility to COVID-19 infection in DM-2 patients using SGLT-2i was not elevated [53]. Regarding ICU admission and MV, Yeh et al. [50] and Perez-Belmonte et al. [45] found no significant associations with prior use of SGLT-2i in DM-2 patients [45, 50]. Dalan et al. [54] suggested a marginally lower risk of MV in DM patients using SGLT-2i [54].

Nevertheless, counterarguments against the widespread utilization of SGLT-2i during the acute phase of infection include the following considerations: SGLT-2i may instigate volume depletion, arterial hypotension, and euglycaemic DKA. The emergence of the latter complication appears notably evident in patients with diminished carbohydrate intake due to acute illness and in those experiencing dehydration from fever, vomiting/diarrhea, and the osmotic/diuretic influences of COVID-19 [92]. Therefore, a judicious evaluation of the appropriateness of SGLT-2i usage is imperative, particularly in individuals with impaired glucose tolerance and subsequent insulin resistance, potentially leading to hyperinsulinism.

### SGLT-2 inhibitors in patients with cardiometabolic risk factors and/or heart failure

SGLT-2i are recognized for their diverse applications, encompassing not only DM-2 but also heart failure, whether with preserved or reduced ejection fraction [4, 93]. Viral diseases, such as COVID-19, have been identified as potential triggers for heart failure [94]. Autopsy studies indicate that SARS-CoV-2 can directly invade cardiomyocytes, leading to direct cardiomyocyte damage [95]. Beyond the virus itself, the inflammatory response, characterized by elevated cytokine levels, contributes to myocardial damage [94].

Hospitalized COVID-19 patients often exhibit elevated levels of creatine kinase (CK) and lactate dehydrogenase (LDH) or increased troponin, indicative of myocardial damage [96–98]. Studies suggest that up to 62.3% of hospitalized COVID-19 patients experience myocardial damage, detectable through elevated high-sensitive cardiac troponin T (Weber). Patients with cardiac injury also demonstrate elevated IL-6 levels, with fulminant myocarditis attributed to a cytokine storm as a potential cause [10]. Preventing cytokine storm appears crucial, given its impact on cardiac function and, consequently, survival.

In addition, COVID-19 may contribute to cardiac remodeling, particularly with a right-dominant pattern. Left ventricular (LV) function is less frequently affected [99]. Viral infections, known to cause subacute myocarditis with limited ventricular function, underscore the importance of SGLT-2 inhibitors in guideline-based heart failure therapy, potentially preventing or reversing cardiac remodeling [75]. However, the impact of COVID-19-induced cardiac remodeling and the potential benefits of SGLT-2i in this context remain to a large extend unclear.

We found that dapagliflozin demonstrates a reduction in cardiovascular events in heart failure patients, and its use before contracting COVID-19 did not elevate severe outcomes. The combination of metformin and empagliflozin protected against COVID-19 hospitalization in those with cardiometabolic risk factors, but empagliflozin alone did not reduce the risk. Experimental evidence suggested dapagliflozin’s potential in mitigating SARS-CoV-2-mediated cardiorenal injury. However, the DARE-19 study found no significant benefits in patients with cardiometabolic risk factors, and a multicountry study reported no association between empagliflozin and COVID-19 outcomes in hospitalized adults [23, 24, 33, 52, 55, 57].

### SGLT-2 inhibitors and acute kidney injury in patients with COVID-19

2021 McMurray and colleagues demonstrated the beneficial effects of SGLT-2i on chronic renal failure progression. In both patients with and without DM, the use of SGLT-2i reduced the risk of progression to chronic renal failure and significantly reduced mortality [4]. Causes of chronic renal failure are diverse. Among others, however, repeated AKI is a possible trigger. Patients with renal function impairment and COVID-19 have increased risk for adverse clinical outcomes, severe disease and higher in-hospital mortality compared to those with normal renal function [100]. In a large veterans’ study from the U.S. in 2021, Al-Aly et al. [101] showed that even 30 days after the initial diagnosis of COVID-19, there was an increased likelihood that renal function was still impaired [101].

The effects leading to improved preservation of residual renal function in chronic renal failure are still unclear [4]. It is suspected that via tubuloglomerular feedback, triggered by the increased, SGLT-2i-induced, flow of sodium through the nephron via adenosine, vasoconstriction of the glomerular arterioles occurs. This leads to protection of the glomeruli by lowering intraglomerular pressure [102]. Stabilization of eGFR occurred during therapy with SGLT-2i and was significantly reduced during follow-up, compared to patients on ACE inhibitors [103]. By stabilizing the progression of chronic renal failure, patients who develop COVID-19 are at less risk of mortality: CKD is associated with higher risk of severe COVID-19 [104].

The relationship between AKI and SGLT-2i in the context of COVID-19 remains less clear. From a pathophysiological perspective, a dual effect is conceivable. The potential protective effect arises from the amelioration of CKD and chronic heart failure. Conversely, the SGLT-2 i-induced volume depletion, leading to an elevated risk of dehydration in infected individuals, may contribute to an increased susceptibility to acute renal injury [105].

We found limited studies on the impact of SGLT-2i on renal function in COVID-19. Dapagliflozin use during COVID-19 in individuals with cardiometabolic risk factors was associated with a 3.4% incidence of AKI in the DARE-19 study [23]. Furthermore, the RECOVERY trial observed no significant difference in AKI occurrence between empagliflozin and standard care groups (6.3% vs. 6.1%, respectively) [57]. However, in a retrospective cohort study, Min et al. [47] reported a notably higher AKI occurrence of 43.4% in patients with type 2 diabetes [47]. Further research is warranted to elucidate the intricate interplay between SGLT-2 inhibitors, COVID-19, and acute kidney injury. The reported variations in AKI incidences among studies examining COVID-19 can be attributed to several factors. Indeed, the differences in COVID-19 severity reflect variations in patient populations across studies. Hospitalized patients, experiencing more advanced disease stages, may significantly impact the observed AKI incidence. Additionally, the inclusion of patients with diverse baseline characteristics and comorbidities contributes to overall variability. Finally, experimental evidence suggests that SARS-CoV-2 induces cardiorenal injury in rats with post-myocardial infarction heart failure and mice with AKI and diabetes by downregulating apelin and ACE2, while upregulating SGLT-2, endothelin-1, and pro-inflammatory cytokines [56].

### SGLT-2i and diabetic ketoacidosis in patients with COVID-19

The infection with SARS-CoV-2 has the potential to directly or indirectly disturb the endocrine system, leading to endocrine dysfunction and dysregulation of glycaemic control. The existing literature on the intricate interplay between COVID-19 and endocrine dysfunctions is continuously evolving and remains incompletely understood [106]. Characterized by hyperglycemia, ketone body accumulation, and resulting acidosis, diabetic DKA is a life-threatening metabolic disturbance. While more prevalent in DM-1, it can also manifest in individuals with DM- 2 during viral infections. Research suggests that COVID-19 can expedite lipolysis, promoting ketosis, particularly in diabetes patients with inadequate glycemic monitoring [107]. Moreover, euglycaemic DKA represents one of the side effects of SGLT-2i, a risk highlighted by FDA drug safety warnings in 2015 [108]. Certainly, observational data emphasize ongoing concerns regarding the incidence of DKA in the use of SGLT-2i [64], particularly in the presence of specific medical conditions such as acute infectious illnesses, urgent surgical procedures, or extended periods of fasting [3]. However, the incidence of DKA in SGLT-2i users during COVID-19 remain unclear.

We found that SGLT-2i, such as dapagliflozin and empagliflozin, are consistently not associated with an increased risk of DKA in patients with DM-2 or those with cardiometabolic risk factors or heart failure during COVID-19, based on four studies [23, 24, 35, 57]. Khunti et al. [35] also reported no higher in-hospital mortality risk with SGLT-2i in DM-2 patients during COVID-19 [35]. However, Min et al. (2022) observed a 9.4% incidence of DKA associated with SGLT-2 inhibitor use in DM-2 patients with COVID-19 [47].

As mentioned above, one significant factor contributing to the disparity in reported DKA incidences is the severity spectrum of COVID-19 within the studied populations. The study of Min et al. [47] specifically focused on hospitalized patients [47]. Severe cases requiring hospitalization may inherently carry a higher risk of metabolic complications, including DKA. This contrasts with studies encompassing a broader spectrum of COVID-19 severities, where the incidence of DKA might be lower due to the inclusion of milder cases managed in outpatient settings. Variations in the severity of COVID-19 can reflect differences in patient populations across studies. Hospitalized patients may exhibit more advanced disease stages, potentially influencing the observed incidence of DKA. Additionally, the inclusion of patients with diverse baseline characteristics and comorbidities can contribute to the variability. Moreover, variations in study designs, including whether the study is retrospective or prospective, and the methods used for data collection, can influence the reported incidence rates. Finally, the focus on hospitalized patients in specific studies may introduce a selection bias toward more severe cases, impacting the overall estimate.

## Area for further research: potential impact of SGLT-inhibitors in the prevention of thrombotic complications in SARS-CoV-2 infection

Initially categorized as primarily affecting the respiratory system, COVID-19 is distinguished by an aberrant immune response and irregular release of cytokines and chemokines, which serve not only to attract immune cells to the site of injury but also to initiate activation of the complement and clotting pathways impacting various organs including the circulatory system [109–111]. Immunothrombosis, a fundamental defense mechanism, involves interactions between the innate immune system, platelets, and endothelial cells, leading to activation of the coagulation system to contain invading pathogens [112], an interplay that appears to be significant in the pathophysiology of severe cases of COVID-19 [113, 114]. Indeed, elevated levels of coagulation factors detected in individuals with COVID-19 likely represent an aspect of hyperinflammatory response [115]. Neutrophils and monocytes release procoagulant substances like IL-1 or tissue factor, as well as serine proteases such as elastase and cathepsin G, which contribute to the augmentation of clot formation [112, 116, 117]. Moreover, enhanced production of tissue factor and diminished functionality of tissue factor pathway inhibitor (TFPI), induced directly by proinflammatory cytokines, resulting in elevated tissue factor levels [118, 119]. In addition, inflammatory molecules have the ability to activate platelets and stimulate their aggregation [120, 121]. The potential role of the kallikrein‐kinin system in driving thrombo‐inflammation in COVID‐19 has also been highlighted arising from its ability to trigger inflammation and coagulation activation through the contact pathway [122–125]. In addition to reducing the levels of thromboregulatory proteins, inflammatory mediators directly hinder the synthesis, activation, and effectiveness of protein C, while promoting its consumption, ultimately leading to immunothrombosis [119]. Among the recognized pathophysiological mechanisms implicated in COVID-19-related coagulopathy, there are hypo-fibrinolytic changes typified by increased production of plasminogen activator inhibitor-1 (PAI-1) by inflammatory agents process, which is accompanied by impaired degradation of PAI-1 due to inhibition of protein C [119, 126]. Conversely, clotting factors, specifically thrombin, have the capability to activate immune cells directly and induce the release of inflammatory mediators [127].

It is assumed that elevated circulating insulin levels with insulin resistance in type 2 diabetes underpins increased ACE-2 expression in lung epithelial cells and, hence, contributes to severe disease associated with COVID-19 infection. Insulin resistance, a hallmark feature of type 2 diabetes, is known to elevate inflammatory cytokines [128], endothelial dysfunction [129] and procoagulant state [128] in this high-risk subgroup even before SARS-CoV-2 infection. Hence, as a result, insulin resistance potentially contributes to the severity of COVID-19 and is associated with poorer outcomes among patients with pre-existing diabetes.

Indeed, in response to SARS-CoV-2, excessive immunothrombosis may precipitate pathological thrombosis in both large and small blood vessels, culminating in significant organ damage and increased mortality rates [130, 131]. The latest is supported by studies showing that critically ill patients exhibit the highest serum concentrations of cytokines, including IL-6, TNFα, IL-1β, IL-8, and IL2R, correlating with the development of ARDS, hypercoagulation, and disseminated intravascular coagulation. This combination could lead to manifestations such as thrombosis, thrombocytopenia, and gangrene of the extremities [132].

Considering the emerging anti-inflammatory properties of SGLT-2 inhibitors with a notable reduction in inflammatory biomarkers, such as C-reactive protein, ferritin, and interleukin-6 and their favorable influence on vascular endothelial function, their utilization may hypothetically confer an advantageous effect in attenuating excessive cytokine production and inflammatory response observed in severe cases of COVID-19 infection with potential relevance in prophylaxis against thrombotic complications associated with SARS-CoV-2 infection [14]. Indeed, in-vitro investigations have revealed that the proinflammatory cytokines TNF-α and IFN-γ induce a significant increase in IL-6 expression in Human Cardiac Microvascular Endothelial cells (HCMEC). This response is markedly suppressed by clinically relevant concentrations of canagliflozin, dapagliflozin, and empagliflozin [14]. Additional experimental studies highlight that the stimulatory effects of TNF-α and IFN- γ on IL-6 expression in Human Umbilical Vein Endothelial cells were significantly attenuated by canagliflozin [14]. Further experimental and clinical studies are needed to explore the potential of attenuating excessive cytokine production and inflammatory response in severe cases of COVID-19 infection, relevant to prophylaxis against thrombotic complications associated with SARS-CoV-2.

## Limitations

Despite our initial intention to conduct a meta-analysis, several challenges were encountered that rendered this approach unfeasible. These challenges primarily stemmed from the heterogeneity observed among the included studies, spanning variations in methodologies, populations, and interventions. The diverse nature of these studies made it challenging and, in some instances, inappropriate to aggregate their findings through a meta-analytical framework. In order to ensure a rigorous and contextually relevant synthesis of the available evidence we adapted our approach to a systematic review.

## Conclusion

In summary, our investigation reveals that individuals using SGLT-2i, both with and without diabetes mellitus, and experiencing COVID-19, demonstrate a noticeable trend towards reduced mortality and hospitalization. Moreover, our findings contribute significant insights into the safety profile of these drugs, suggesting a low risk of adverse events, such as AKI and DKA, even in acute settings when appropriately administered. Additionally, our results may serve as a foundation for potential future applications of this drug class in preventing organ damage and reducing cardiovascular events among hospitalized patients, irrespective of their diabetes status and COVID-19 presence. Nevertheless, it is imperative to acknowledge that this hypothesis necessitates rigorous testing through adequately designed and powered clinical studies.

### Supplementary information

Supplementary Information
