# Supplementary material for: Potential use of sodium glucose co-transporter 2 inhibitors during acute illness: a systematic review based on COVID-19
Source: Endocrine. 2024 Mar 6;85(2):660–75. doi: 10.1007/s12020-024-03758-8 (PMC11291544; doi:10.1007/s12020-024-03758-8)
Supplement: Supplementary file 1 — Supplementary Information [file 12020_2024_3758_MOESM1_ESM.doc]

# **Table S1.** Quality Assessment of used studies – Newcastle-Ottawa Quality Assessment Scale for Cohort Studies

| Study | Selection | Comparability | Exposure | Score |
| --- | --- | --- | --- | --- |
| Sourij et al., 2020 | **🟑🟑🟑🟑** | **🟑🟑** | **🟑🟑🟑** | **9** |
| Wander et al., 2021 | **🟑🟑🟑** | **🟑** | **🟑🟑🟑** | **7** |
| Luk et al., 2021 | **🟑🟑🟑🟑** | **🟑🟑** | **🟑🟑🟑** | **9** |
| Ramos-Rincon et al., 2020 | **🟑🟑🟑** | **🟑🟑** | **🟑🟑🟑** | **8** |
| Silverii et al., 2020 | **🟑🟑🟑🟑** | **🟑** | **🟑🟑🟑** | **7** |
| Kim et al., 2020 | **🟑🟑🟑🟑** | **🟑🟑** | **🟑🟑🟑** | **9** |
| Khunti et al., 2021 | **🟑🟑🟑🟑** | **🟑🟑** | **🟑🟑🟑** | **9** |
| Elibol et al., 2021 | **🟑🟑🟑🟑** | **🟑🟑** | **🟑🟑🟑** | **9** |
| Israelsen et al., 2021 | **🟑🟑🟑🟑** | **🟑🟑** | **🟑🟑🟑** | **9** |
| Kahkoska et al., 2021 | **🟑🟑🟑🟑** | **🟑🟑** | **🟑🟑🟑** | **9** |
| Shestakova et al., 2022 | **🟑🟑🟑🟑** | **🟑🟑** | **🟑🟑🟑** | **9** |
| Kosiborod et al., 2021 | **🟑🟑🟑🟑** | **🟑🟑** | **🟑🟑🟑** | **9** |
| Mancia et al., 2020 | **🟑🟑🟑🟑** | **🟑🟑** | **🟑🟑🟑** | **9** |
| Sainsbury et al., 2021 | **🟑🟑🟑🟑** | **🟑🟑** | **🟑🟑** | **8** |
| Pérez-Belmonte et al., 2020 | **🟑🟑🟑🟑** | **🟑🟑** | **🟑🟑** | **8** |
| Min et al., 2022 | **🟑🟑🟑🟑** | **🟑** | **🟑🟑🟑** | **8** |
| Salgado-Barreira et al., 2023 | **🟑🟑🟑🟑** | **🟑🟑** | **🟑🟑🟑** | **9** |
| Dalan et al., 2021 | **🟑🟑** | **🟑🟑** | **🟑🟑🟑** | **8** |
| RECOVERY Collaborative Group, 2023 | **🟑🟑🟑** | **🟑🟑** | **🟑🟑🟑** | **8** |
| Sandhu et al., 2023 | **🟑🟑🟑🟑** | **🟑🟑** | **🟑🟑🟑** | **9** |
| Bhatt et al., 2023 | **🟑🟑🟑** | **🟑🟑** | **🟑🟑🟑** | **8** |
| Foresta et al., 2023 | **🟑🟑🟑🟑** | **🟑🟑** | **🟑🟑🟑** | **9** |

# References

Wells GA, Shea B, O'Connell D, et al. The Newcastle-Ottawa Scale (NOS) for assessing the quality of nonrandomized studies in meta-analyses. [http://www​.ohri.ca/programs​/clinical_epidemiology/oxford.asp](http://www.ohri.ca/programs/clinical_epidemiology/oxford.asp). Accessed January 11, 2016.
